# Notes

**Published:** 1898-03-19

**Authors:** 


### NOTES.

H.R.H. the Duke of York has promised to be a vice-president of the Royal National Hospital for Consumption, Ventnor.

Dr. W. J. Simpson, D.P.H., late Health Officer of Calcutta, has been appointed to the Professorship of Hygiene at King's College, London.

The hospital at Bulawayo is very full, this being the "fever season," and it has been necessary to requisition the services of additional nursing sisters. An extension of the hospital is shortly to be made at a cost of £6,000.

At the opening ceremony of the new Victoria Cottage Hospital at Accrington, special thanks were tendered to Miss Poole, matron of the Blackburn Infirmary, for her valuable suggestions as to the finishing, furnishing and fitting of the hospital.

Invalidism has its profitable side. Many speculators who invested in a contingent of seats for Miss Farren's benefit at Drury Lane are advertising to this effect: "A widow lady who contributed her mite for Miss Farren's benefit, has been rendered so ill by the excitement of the prospect of going to a theatre, that she is obliged to part with the ticket, &c." This is a variation on the many money-making uses to which influenza has been put.

During the mayoralty of Sir George Faudel-Phillips £657,000 was raised at the Mansion House for the relief of the sick, afflicted, and famine-stricken.

A new Workhouse Hospital has just been opened at Rochdale, which is intended to provide temporary accommodation for the sick, pending the erection of the new infirmary of 300 beds which the Guardians have decided to build.

During her sixty years' reign the Queen has given 6,100 guineas to the Seamen's Hospital at Greenwich. The authorities of this hospital are always grateful for gifts of cash, clothing, knitted scarves, cardigans, &c., for their seafaring patients.

The Jewish community of Bombay have opened a plague hospital of 16 beds, and a segregation camp, for the exclusive use of sufferers from the plague who belong to the Beni-Israel sect. The hospital was dedicated by the reading of the special prayer for use in time of plague from the Maamadoth.

In the new diphtheria ward added to the Delancey Hospital, Cheltenham, there are no mantelpieces whereon dust may collect. Each bed has an adjustable bed-table and there is an electric button over every bed, with a handle so arranged that the patient may press the button without moving his position.

The *Medical News* of New York says that the day-book of the chief medical officer of the cruiser *Maine* has been recovered from the wreck. It bears witness to the methodical habits of Surgeon Honeberger, for the record was brought up to the last moment before the wreck of the ship.

The annual report of the London and Counties Medical Protection Society, which will be presented to the general meeting to be held on March 29th, shows another year of useful work. A large number of important cases have been dealt with successfully, and in the majority it has been found possible to defend members with complete success without incurring the heavier expenses which would have been involved had the cases gone into court. Complaints from members of slanders uttered against them have been numerous, and in many cases ample apologies have been exacted. The income of the society for the past year has been £746, and the guarantee fund represents more than £2,000.

At the last ordinary meeting of the council of the Royal College of Surgeons it was stated that, since December 3rd, the laboratories had supplied to the Metropolitan Asylums Board 7,275 doses of antitoxic serum, and that the demands of all the hospitals had been fully met. Antitoxin had also been supplied to various general and children's hospitals as well as to certain medical practitioners. A petition was received signed by certain Fellows and members of the college claiming that the right of electing a representative of the college to the General Medical Council by section 7 of the Medical Act, 1886, vested in the Fellows and members of the college, and requesting the council to forthwith make arrangements to hold an election and enable the Fellows and members to choose a representative. It was resolved that the petitioners should be informed that the questions raised by them were under consideration.

The Prince of Wales's Hospital Fund has received the sum of £672, the balance of the amount resulting from the exhibition of the Queen's Diamond Jubilee presents at the Imperial Institute.

The Lord Mayor's Fund for providing a home of rest for women as a memorial to the late Duchess of Teck is progressing well, and a ladies' committee has just been formed.

A very welcome gift of £10,000 has been made to the Prince of Wales's Hospital Fund by the Peabody Trust Fund, on the ground that it is a matter of great importance to the persons for whom the Trust was created that adequate hospital accommodation should exist in the metropolis.

The Lord Mayor has remitted to the Mayor of Maidstone a cheque for £927 8s. 9d., the balance of the Mansion House Fund for the relief of the sufferers through the typhoid epidemic. The Mayor's Fund at Maidstone is still open, and funds are badly needed for the widows and orphans' branch of the Fund.

It is stated that the Maidstone Water Company is about to have a combined suit brought against them by several hundred recent typhoid sufferers to recover damages for the loss suffered by them through the epidemic. The water company's offer of £3,000 as compensation for injuries received has been rejected. Sanitarians will feel much interest in the result of this novel law suit.

